# Bio- and Nanotechnology as the Key for Clinical Application of Salivary Peptide Histatin: A Necessary Advance

**DOI:** 10.3390/microorganisms8071024

**Published:** 2020-07-10

**Authors:** Carolina Reis Zambom, Fauller Henrique da Fonseca, Saulo Santesso Garrido

**Affiliations:** Department of Biochemistry and Organic Chemistry, Institute of Chemistry, UNESP-Sao Paulo State University, Araraquara/SP 14800-060, Brazil; fauller.henrique@unesp.br

**Keywords:** *Candida albicans*, antifungal peptides, nanoparticles, histatin-5

## Abstract

*Candida albicans* is a common microorganism of human’s microbiota and can be easily found in both respiratory and gastrointestinal tracts as well as in the genitourinary tract. Approximately 30% of people will be infected by *C. albicans* during their lifetime. Due to its easy adaptation, this microorganism started to present high resistance to antifungal agents which is associated with their indiscriminate use. There are several reports of adaptive mechanisms that this species can present. Some of them are intrinsic alteration in drug targets, secretion of extracellular enzymes to promote host protein degradation and efflux receptors that lead to a diminished action of common antifungal and host’s innate immune response. The current review aims to bring promising alternatives for the treatment of candidiasis caused mainly by *C. albicans*. One of these alternatives is the use of antifungal peptides (AFPs) from the Histatin family, like histatin-5. Besides that, our focus is to show how nanotechnology can allow the application of these peptides for treatment of this microorganism. In addition, our intention is to show the importance of nanoparticles (NPs) for this purpose, which may be essential in the near future.

## 1. Introduction

*Candida* spp. are reported as the most common fungal pathogens with the ability to cause superficial infections to progress to systemic infections in human hosts, especially those who are immunocompromised [1]. This class of microorganism is responsible for major systemic fungal infections representing high mortality rates and generating expressive healthcare costs worldwide [2]. *Candida albicans* is the most important species because it is more frequently isolated in humans and cause ~1.5 million deaths per year around the world [3]. In addition, this species is quite versatile, being able to develop in environments with different conditions such as different nutrient availability and different oxygen rates, which demonstrates an incredible ease of adaptation [4].

As a commensal pathogen, *C. albicans* is able to change its morphology from yeast to hyphae and become more infectious depending on the host’s immune response. This set of characteristics has a direct influence on the virulence of this species, which has been increasing progressively over the years [5]. The hyphae morphology represents an important virulence factor for this pathogen [6] besides the ability to form biofilm. Those characteristics make this species even more difficult to eradicate [2].

Nevertheless, there are several reports of adaptive mechanisms that this species can present when exposed to conventional antifungal agents, such as fluconazole. Some of these mechanisms are related to stress responses, intrinsic alteration in drug target and enzymatic degradation that permit this microorganism to survive even during antibiotic treatment [7]. For this reason, candidiasis caused by *C. albicans* is currently prevalent, especially in hospitalized patients. Medical interventions such as chemotherapy for cancer treatment and immunosuppression for transplantation makes the patient more susceptible to *C. albicans* infections [8].

Therefore, the search for new antifungal drugs is very important and must be constantly developed. A promising alternative is the use of bioactive peptides which naturally participate in innate immune response of the human body. An example of this are the Histatin peptides found in human saliva. This histidine-rich class of peptides presents antifungal activity in addition to playing an important role in wound healing and oral re-epithelization [9]. Histatin-5 is the most potent antifungal peptide in the Histatin family [10] and has a wide action against pathogenic fungi, including *C. albicans*.

However, this peptide has not yet been used in therapy. One reason for this is its enzymatic degradation when it is present at the site of action. Therefore, the purpose of this review is to highlight some mechanisms that confer resistance to *C. albicans* and the importance of this peptide in the eradication of this microorganism. We described here what has been developed in the last three years regarding this peptide and its use. In addition, this review proposes the use of nanotechnology as an important tool that can facilitate the clinical use of this peptide. In fact, the use of nanotechnology and drug delivery systems can lead to a step forward in the field of antifungal therapy to allow its use without the risk of enzymatic degradation, increasing its time of residence at the site of action and providing a prolonged and non-cytotoxic treatment.

## 2. *Candida albicans*: Virulence Factors and Resistance

*Candida albicans* is a diploid polymorphic fungus that can switch between the yeast and hyphal growth modes and exists as a common member of the human microflora, asymptomatically colonizing the skin and the gastrointestinal, respiratory and genitourinary tracts as a commensal microorganism [11,12]. The commensal state of *C. albicans* is maintained by some factors as intact epithelial barriers, innate immune system responses and coexisting microflora, but in situations in which these factors are absent or weakened, *C. albicans* proliferation is favored and this fungus can become an opportunistic microorganism, progressing from superficial infections to systemic infections that can lead to death [12,13]. Risk factors for *Candida* spp. superficial or systemic infections can be divided in three categories: (I) factors that promote colonization by *Candida*, for example broad-spectrum antibiotics therapy; (II) factors that cause immunosuppression, such as organ transplant, HIV, neutropenia, use of chemotherapeutic agents, age, malnutrition; and (III) factors that provide a route for *Candida* infection, for example, surgical procedures, parenteral nutrition, hemodialysis, mechanical ventilation and catheters [14,15].

In order for *C. albicans* to colonize and persist in a host, as a commensal or as a pathogen, its adhesion to a mucosal surface is essential. The first step is overcoming the mucous barrier that protects the host epithelium in order to access it and promote a physical interaction between the epithelial cells and the fungus [16,17,18]. The initial interaction is thought to involve the yeast form, but studies in murine models showed that yeast and hyphal morphologies can be observed in the gastrointestinal tract during commensal colonization [18,19]. Both morphologies have developed mechanisms to promote the adhesion to the epithelial tissue; the adhesins proteins are the most important ones. The agglutinin-like sequence (Als) proteins Als5p and Als3p mediate the adhesion of yeast cells and hyphal form to the host epithelium, respectively [20,21]. These proteins are linked to the β-1,6-glucans present in the fungal cell wall and, along with other proteins of the Als family, are responsible for recognizing and binding to different surfaces, from epithelial tissues to abiotic surfaces, such as dentures and medical devices [22]. Besides the importance of this family of protein for *C. albicans* adhesion to the host epithelial tissues, they also participate in the adhesion of other microorganisms to *C. albicans* hyphae, like other *Candida* species and *Streptococcus oralis* [23,24]. Furthermore, the amyloid forming region (AFR), located in the N-terminal domain of Als proteins, allows the interaction between Als of different cells, which is important for the aggregation of *C. albicans* cells and hyphae [25].

Once *C. albicans* is adhered to the host epithelium, there are two mechanisms by which it can be internalized. The first process, which allows internalization of viable and nonviable fungi, is called induced endocytosis. It is initiated by the interaction between fungal proteins, like the invasin Ssa1p or adhesin Als3p, and host E-cadherin or EGFR/Her2 complexes. This binding activates a chlatrin-dependent mechanism that induces actin cytoskeleton remodeling, producing pseudopods responsible for pulling the fungus into the host cells [26,27,28]. The second process, called active penetration, requires metabolically viable fungi. It is a combination of the mechanical tension caused by the elongation of hyphal tips and the action of secreted hydrolytic enzymes and cytolytic peptides, which facilitate extension of the hyphae through or between epithelial cells [29,30]. *C. albicans* is known to secrete 255 enzymes that act directly as virulence factors, but two classes of extracellular enzymes have a major role in adherence, tissue invasion and nutritional acquisition by *C. albicans*, aspartyl-proteinases (SAPs) and phospholipases [28,31,32]. The SAPs secreted by *C. albicans* have the ability to degrade a broad-range of host proteins: Sap2p and Sap5p are related to degradation of E-cadherin and mucins present in the gastrointestinal epithelium, facilitating hyphae penetration [33,34]. These proteinases are also involved in the degradation of host defense factors, like antibodies and antimicrobial peptides [16,35]. For example, it was observed *in vitro* that the killing ability of the peptide histatin-5, in salivary concentrations, was inversely proportional to *C. albicans* cell density, due to degradation by Sap9p [36]. The cell surface protein Msb2p is also responsible for degrading antimicrobial peptides, such as histatin-5 and LL-37 [37]. Moreover, Moyes et al. identified a cytolytic peptide, secreted by *C. albicans* only in the presence of epithelial cells. The *C. albicans* hyphal-specific cell elongation 1 gene codes for the protein Ece1p, comprised by 271 amino acids, which is cleaved by Kex2 protease into eight secretory peptides, being the fungal cytolytic peptide one of these peptides. This peptide was called candidalysin and it was pointed by the authors as the most important fungal cytolytic peptide toxin in the process of mucosal infection by *C. albicans* [38]. The peptide has the ability to permeabilize epithelial cell membranes, damaging it through a “carpet-like mechanism”, that leads to enzymatic and ionic effluxes, besides stimulating cytokine secretion [38]. Besides the importance they have for *Candida* pathogenicity, the invasion and colonization of the human tissue by some species of *Candida* spp. may also be a possible trigger for autoimmunity issues. There are some reports of celiac disease (CD) [39], food allergy [40] and inflammatory bowel diseases (IBD) associated with *C. albicans* infections [41]. Some hypotheses describe that *C. albicans* presents hyphal wall proteins that show similarities with T-cell gliadin epitopes [42]. The protein Hwp1, expressed by *C. albicans*, presents sequence analogy with the gluten protein gliadin. Due to the Hwp1 protein being also a substrate for the transglutaminase enzyme, some cases of *C. albicans* infection could be associated with Celiac disease [42]. In addition, it was also reported that microorganisms like *C. albicans* can remodel some immune cells to induce memory and Trained Immunity (TRIM) associated with some types of food allergy. In this case, β-glucan, an integral cell wall component of *C. albicans*, can lead to TRIM mediated activation of dectin-1 receptor and the mTOR pathway [40].

The ability of *C. albicans* to form biofilms also has clinical significance. Biofilms are surface-associated microbial communities, which show increased expression of virulence and drug-resistance factors, like adhesins and efflux pumps, leading to alterations in stress response and decreased susceptibility to phagocytosis when compared to the microorganism planktonic form [32,43]. Besides the drug-resistance factors, the extracellular matrix is also responsible for preventing bioavailability of antifungal drugs [44]. *Candida* biofilms can be associated to abiotic surfaces, like medical devices, where they can lead to the development of bacterial infections by *Staphylococcus aureus* and *Pseudomonas aeruginosa* in the case of mechanical ventilation, for example [45]. 

Regarding the oral cavity, it is possible to observe the formation of monomicrobial and polymicrobial biofilms on the oral epithelium or dentures surfaces, for example [46]. Dental caries is the most common oral disease and is associated to the bacteria *Streptococcus mutans*. Evidence points to *C. albicans* having a potential role in dental caries, since dental biofilms of associated *S. mutans-C. albicans* are commonly found. Moreover, both species are able to produce and tolerate acid compounds responsible for damaging the tooth [47,48,49]. Some works also exploited the association of *C. albicans* to *Streptococcus oralis*. In vitro studies conducted by Cavalcanti et al. showed greater levels of filamentation and biofilm development by *C. albicans* when *S. oralis* was present [50]. Xu et al. observed facilitated *C. albicans* invasion in mucosal tissue, mouse tongue and kidney when associated to *S. oralis*, related to an increase in the degradation of E-cadherin by calpain [51]. *S. oralis* was also shown to induce expression of *Candida* genes and proteins that promote bacteria adherence to fungal hyphae [24]. *Streptococcus gordonii* is also capable of forming biofilms in association to *C. albicans*, which provides resistance to antimicrobial treatment for both microorganisms [52,53]. In contrast, the interaction between *C. albicans* and the species *Pseudomonas aerugionosa* or *Fusobacterium nucleatum* seems to be antagonistic. Studies involving *P. aerugionosa* showed inhibition of *Candida* hyphae development [54]. The interaction between *C. albicans* and *F. nucleatum* reduced the ability of both the microorganisms to escape from phagocytosis [55,56]. These data show the importance of inter-species interactions and microbial equilibrium in the maintenance of good oral health.

The first line of defense against *C. albicans* are the oral epithelial cells which not only act as a physical barrier, but also can minimize adherence, by processes like desquamation, upon recognizing *C. albicans* hyphae [57,58]. Saliva also represents an important factor on preventing *C. albicans* colonization and adherence, either by mechanical removal of *Candida* cells or by the many antimicrobial peptides (AMPs) in its composition [57,59]. The most outstanding peptides related to *Candida* control in the oral cavity are the histatins, defensins, cathelicidins and lactoferrin [60]. The first ones are a family of histidine-rich cationic peptides, part of the defensins group, which will be better described ahead. Defensins are cysteine-rich peptides produced by neutrophils (α-defensins) and epithelial cells (β-defensins) [61]. There is only one human cathelicidin known, called LL-37, that impedes *Candida* adhesion by interacting with cell wall components of the fungus and, additionally, is capable of inducing chemokines [62]. Finally, lactoferrin has the ability to disrupt the fungal plasma membrane and alter iron availability within the fungal cell [63,64]. The Th1 and Th17 lymphocytes are involved in adaptive immunity, and the last ones are suggested to be responsible for long-term immunity to oral mucosal infections [58,65,66]. Those facts are associated with a greater incidence of oral-related *C. albicans* infections on immunodeficient or immunosuppressed patients. For instance, studies reported higher levels of the *C. albicans* specifically IgA in HIV+ individuals, who show lower levels of the peptide histatin-5 and depleted Th17 lymphocytes [67,68].

In terms of therapeutic strategies to treat fungal infections, azoles, echinocandins and polyenes are available to treat systemic infections, while all the antifungals available in the market to treat mucosal candidiasis target the ergosterol present in the cell membrane [69,70]. The extensive and frequent use of antifungals as prophylactic or therapeutic treatments of humans and animals, in agriculture, wood and textile industries, led to the development of multidrug-resistant strains of medically relevant fungi, being the resistance to azoles and echinocandins the most reported [71,72,73]. While echinocandins show fungicidal activity against most of *Candida* species, azoles are fungistatic, providing the possibility of acquired resistance development [74,75]. The prevalence of fluconazole resistance in *C. albicans* is low, but higher rates are observed for *Candida glabrata* with rates up to 13%, for *Candida auris* which rates near 93% resistance, and *Candida krusei*, which has shown innate resistance [76,77,78]. Some of the reported resistance mechanisms involve amino acid substitutions on the molecular targets of the antifungals—like that observed in the Fks subunits of glucan synthase, target of the echinocandins [79]—or in the enzyme 14-alpha demethylase, targeted by azoles [80,81]. Metabolic upregulation and overexpression of genes also contribute to resistance mechanisms. The increased chitin synthesis was observed for echinocandin-resistant *Candida* species; in the same way, overexpression of the ERG11 gene, encoding for the enzyme 14-alpha demethylase, is associated to azole-resistant fungi, along with other mutations [8,82]. Mutations in the gene ERG3 resulting in a loss of function of the enzyme sterol Δ^5,6^-desaturase lead to cross-resistance to azoles and polyenes, since the cell circumvents the production of toxic methylated sterols and also accumulates alternative membrane sterols to replace ergosterol [83,84]. Finally, efflux transporters are a relevant feature in the resistance mechanisms, since they do not allow the drug to accumulate intracellularly. In *Candida* species, three proteins are related to fluconazole resistance: the ATP-dependents *Candida* drug resistance proteins 1 and 2 and the proton-motive force- driven protein Mdr1p [85,86,87].

The emergence of resistant *Candida* species leads to a difficulty to find new drugs with absence of host toxicity [70]. One way to face this obstacle are the antimicrobial peptides (AMPs) that are part of the innate defense mechanisms of the host and could play an important role in the fight against fungal infections.

## 3. Histatins and Their Role in the Antifungal Treatment

Several AMPs play an important role in the innate immune response of living organisms. These peptides are present in all human systems and tissues and continuously protect against fungi, bacteria, viruses, protozoa and other parasites [88]. Peptides such as α and β defensins, cathelicidins and histatins are part of the Antifungal Peptides (AFPs) which widen antimicrobial action [89].

The peptides of the Histatin family are the most abundant group of proteins present in human saliva. They are histidine-rich peptides secreted by the parotid and submandibular glands and were first described by Oppenheim et al. [9]. These peptides have a strong antifungal and antibacterial activity against many oral pathogens species and are important for the maintenance of the oral homeostasis, helping in wound healing and oral re-epithelization [90,91]. Histatin-1, -3 and -5 are the three major histidine-rich peptides present in human saliva [9]. Histatin-5 is a proteolytic product of histatin-3 and is the peptide with the greatest antifungal action in the Histatin family. This peptide has 3 to 4 KDa, a sequence of 24 amino acid and positive charge in physiological pH. This peptide assumes an α-helix structure in DMSO (dimethylsulfoxide) and in TFE (trifluorethanol)/water, but when in water it assumes a random structure [92]. Histatin-5 has a wide antifungal action against pathogenic fungi, including *Candida albicans* [93] and *Candida glabrata* [94]. Recent studies have shown that histatin-5 is able to inhibit the growth of *C. albicans* strains in concentrations from 25 to 800 μg mL^−1^ [95], with a minimum inhibitory concentration (MIC) of 8–16 μg mL^−1^ [96].

Although its mechanism of action is not fully understood yet, studies indicate that the ATP efflux and oxidative stress caused by interaction with mitochondria cells lead the microorganism to death [10,90,97]. In addition, studies have shown that the targets of this peptide are intracellular and unlike others antimicrobial peptides, pore formation and membrane lysis caused by histatin-5 have been disproved. Histatin-5 is able to penetrate the cell of *C. albicans* through the membrane receptor Ssa 1/2 and glycans and its internalization mechanism is energy dependent [11]. This membrane receptor on *C. albicans* cells works as a cell market for its action and makes this peptide selective without cytotoxicity for human cells [98]. 

A recent study conducted by Basso et al. showed that the ATP efflux caused by histatin-5 is slow and prolonged and leads to a slower cell death compared to the peptide RTD-1 used in this study [99]. Despite that, recently Pathirana et al. proved the effectiveness of this peptide in inhibiting seven resistant strains of *C. auris*, a multidrug resistance microorganism. Histatin-5 inhibited from 55 to 90% of *C. auris* cells in a dose of 7.5 µM [100]. However, it is not only against fungi that this peptide has antimicrobial activity. Du et al. demonstrated that histatin-5 is able to inhibit multi-drug resistance ESKAPE pathogens (*Enterococcus faecium*, *Staphylococcus aureus*, *Klebsiella pneumoniae*, *Acinetobacter baumanni*, *Pseudomonas aeruginosa* and *Enterobacter species*) [101]. Only 30 µM of histatin-5 was enough to inhibit five of the six tested pathogens. Differences in the mechanism of action of this peptide were also found among these pathogens. For *E. faecium* and *E. cloacae*, the peptide needs to be internalized by an energy-dependent manner; for *p. aeruginosa* and *A. baumanni*, the rapid antibacterial action is related with membrane pore formation and lysis [101]. This result, together with others described here, proves that this peptide has broad antimicrobial activity and multiple mechanisms of action.

In 2014, Puri and Edgerton proposed that histatin-5 antifungal activity could also be related to its ability to bind metals [97] with two specific regions preferred for metal binding. The N-terminal region shows an ATCUN motif binding, in which Cu (II) and Ni (II) can be connected. And the C-terminal region contains Zn (II) binding motif [102]. The study conducted by Conklin et al. demonstrated that the binding with Cu (II) can improve the antifungal action of the peptide showing an IC_50_ below 5 µM. The same study also showed that the first 12 amino acids play an important role in binding to Cu(II) [103]. In addition, a connection with Fe (III) was also reported, and this metal was able to induce α-helix formation, but this is not related to an antifungal activity increase [104].

Even though this peptide shows several mechanisms of action and several options to enhance its antifungal activity, resistance in some *C. albicans* strains against histatin-5 has been reported. An example of this mechanism is related to histatin-5 proteolytic cleavage by aspartic proteases as well as the linkage with the membrane protein Msb2 that prevents its internalization [36,105,106]. Li et al. also demonstrated that polyamine efflux transporters, like FLU1, are able to cause the efflux of histatin-5, preventing its antifungal action [107]. However, that study also noted that the appearance of the mutation that leads FLU1 to promote histatin-5 efflux is not caused by overexposure to this peptide. Hampe et al. also noted the same when investigating transcription factors that regulate FLU1 expression [108]. Although the FLU1 mutation is not associated with overexposure to histatin-5, it is associated with overexposure to fluconazole. It is interesting to note that excessive exposure to conventional drugs promoted not only resistance to these treatments, but also resistance to an innate host defense mechanism such as the peptide histatin-5 [108].

As already mentioned in this review, the emergence of resistant strains brings up a serious public health problem due to the existence of only four classes of drugs available for treatment (azoles, echinocandins, polyenes and flucytosine) [109]. Even though there have been reports that some strains of *C. albicans* are resistant to histatin-5, this molecule is still attractive for clinical treatment against resistant pathogens. The advantage of using AFPs is the possibility of modifying their amino acid sequence to create analogues and hybrids. These modifications could improve their antifungal activity and also allow the peptide to avoid the pathogen resistance mechanisms.

Dh-5 was one of the first histatin-5 analogs to be produced. Its amino acid chain corresponds to the C-terminal sequence of histatin-5 known to be responsible for the antifungal action (residues 11–24) [110]. However, this peptide did not show an increase in its activity and for this reason the dhvar1 and dhvar2 analogues were produced. The amino acid sequence of these peptides was modified in order to increase their hydrophobicity and to assume an α-helix structure. These modifications promoted a 6-fold increase activity by showing a pore formation mechanism of action [110,111,112]. This mechanism of action differs these analogues from the original peptide histatin-5, which has no membranolytic action. Thus, this hydrophobic characteristic allows these analogues to act against other pathogens such as *Torulopsis glabrata*, *Prevotella intermedia*, *Streptococcus mutans*, and methicillin-resistant *Staphylococcus aureus* [111,113].

Besides modifying the amino acid sequence, another widely used strategy to improve peptide properties is the fusion between two different peptides. Lu et al. produced the chimeric peptides cCF10-dhvar4 and cOB1-dhvar4 that showed an LC_50S_ of 1 µM, 10 times greater than the original dhvar4 [114]. Another example of this would be the combination of histatin-5 and halocidin demonstrated by Han et al. [96]. This combination produced three hybrid peptides named di-PH2, di-WP2 and HHP1, which showed MICs of 1–2, 2–4 and 2–4 μg mL^−1^, respectively [96]. The hybrid peptide di-WP2 did not present cytotoxicity and maintained its action even in a medium with 150 mM NaCl [96]. Results like this demonstrate that the fusion of two different peptides results in an improvement not only in the antifungal activity of histatin-5, but also in the gain of other functions provided by the union of two different molecules. However, it is not only the increased antimicrobial action that the production of hybrid or chimera peptides can provide. By joining two fragments or two different peptides, the result can produce a new molecule that has a dual-effect or synergic effect. The hybrid peptide DR9-RR14 produced by Basiri et al. joined the active fragment of histatin-3 with the active fragment of statherin, resulting in a peptide with the potential to prevent tooth demineralization and antimicrobial activity [115].

Perhaps the most important advantage provided by the production of histatin-5 hybrids and analogues is to avoid resistance mechanisms showed by *C. albicans*. Ikonomova et al. proposed the replacement of two lysines (positions 11 and 17) for two arginines in the histatin-5 sequence. This modification increased the antifungal activity and provided resistance to *C. albicans* proteolytic cleavage [116,117]. The same result was observed by Jephthah et al. when producing a hybrid peptide of histatin-5 and spermidine. The Hst4-15-Spd peptide showed an increase in its antifungal activity in addition to resistance to aspartic proteases secreted by *C. albicans* [118]. As described in this topic, there are countless histatin-5 analogs already produced and with proven effectiveness for inhibiting *C. albicans* growth. The analogues mentioned here, and their amino acid sequence, can be seen in Table 1. The produced analogues show extremely important characteristics for their clinical use, such as a low dose of action and non-cytotoxicity. However, they are not yet used in therapy, and there are only few reports of in vivo studies.

One reason for this is that histatin-5 and its analogs are susceptible to enzymatic degradation when used in the human body. Moffa et al. found that histatin-5 is the major portion of the glandular proteins but disappear rapidly when in the whole saliva [119] and the cause of this difference is based on the proteolytic cleavage. Thus, its action is reduced or inactivated and its use to treat fungal infections in the oral cavity is difficult. In summary, to make possible the use of this peptide and its analogs, it is necessary to develop strategies that allow its application at the site of action. Such a strategy should protect the peptide against enzymatic degradation and provide a longer residence time at the site of action. As suggested in some studies, the use of nanotechnology to produce protein delivery systems can ensure the biological functions of histatin-5 and its analogues and promote its use for clinical treatment.

## 4. Nanoparticles as a Strategy to Allow Histatins’ Clinical Application

For the last 10 years, the advancement of nanotechnology has allowed the emergence of nanoparticles (NPs), that are able to incorporate bioactive molecules and direct their delivery to the site of action. NPs can be defined as ultra-dispersed supramolecular structures, which range in size from 10 to 1000 µm [121]. The drug can be encapsulated, dissolved, trapped or attached to the structure of the nanoparticle, which plays an important role in the delivery of drugs in clinical applications. The use of NPs can improve solubility, decrease toxicity, increase therapeutic doses without a risk of side effects, increase bioavailability and residence time at the action site and protect drugs against enzymatic degradation [121]. For this reason, the use of NPs is advantageous and can allow the application of AMPs with therapeutic action in clinical treatments. 

An example is the liposome loaded with peptide LL-37, which provided a reduction in its cytotoxicity and increased its effectiveness as an antiviral, being promising for the treatment of HSV-1 in humans [122]. The use of liposomes loaded with the peptide ghrelin promoted its protection against enzymatic degradation by trypsin and carboxylesterase [123], and allowed the nasal application of this peptide to treat cachexia, an extreme weakness that affects hospitalized patients with chronic diseases. Other NPs have also been used to protect and improve the action and bioavailability of AMPs. Polymeric NPs produced with cellulose, chitosan and poly(lactic-co-glycolic) acid (PLGA) were used to protect peptides and proteins from degradation by digestive enzymes [124]. Perfluorocarbon NPs loaded with thrombin inhibitor peptide improved its antithrombotic activity in vivo [125]. Chitosan NPs loaded with nisin increased its antimicrobial activity against *S. aureus* and *Listeria monocytogenes* [126]. There are many other examples that prove that the use of NPs loaded with peptides is a strategy capable to promote their therapeutic use.

However, NPs are rarely used with peptides and the few drugs available are in the early stages of development [124]. There are even fewer studies involving NPs and peptides from the histatin family. These peptides have a great antifungal action and play an important role in regulating oral homeostasis, but, as already described in the previous item, they undergo enzymatic degradation at their site of action. For this reason, the combination of these peptides with NPs emerges as a strategy to prevent them from degradation and to increase the residence time in their site of action.

Although there are not many reports of the incorporation of these peptides in NPs, there are recent reports of the use of histatin-1, -5 and some of their structural analogues being incorporated into mucoadhesive hydrogels. The incorporation of these peptides in hydrogel allows their application in the oral mucosa and helps prevent and treat oral candidiasis. Their incorporation also promotes the re-epithelialization and wound healing in the oral cavity [127,128,129]. Lin et al. incorporated histatin-1 in chitosan hydrogel and observed a prolonged release that generated increased cell adhesion, migration and angiogenesis, important factors for wound healing [127].

The incorporation of histatin-5 in hydroxypropyl methylcellulose (HPMC) hydrogel demonstrated efficacy for the treatment of oral candidiasis in vivo. The HPMC hydrogel showed slow release with rapid antifungal action [128]. The same HPMC hydrogel was used to incorporate K11R-K17R, an analogue of histatin-5 with superior antifungal activity. The hydrogel was effective in inhibiting resistant strains of *C. albicans* [129]. The wide action of this new analogue was demonstrated through its incorporation in HPMC hydrogel and use for the treatment of denture stomatitis in vivo. The formulation was effective to prevent biofilm formation in denture devices [130]. This result is important since as patients who wear dentures are affected by *C. albicans* repeatedly. The infection by *C. albicans* happens because it is common for the microorganism to form biofilms and colonize the surface of the denture, which becomes a source of contamination for the palate tissue, even after treatment with conventional antifungals.

Thus, the complete eradication of the microorganism from the denture is extremely important, as this is the only way to avoid recolonization [130]. In the search for a way to prevent the colonization of *C. albicans* in the denture, Wen et al. developed a material composed by layers of histatin-5 alternated with hyaluronic acid [131]. With its outermost layer composed of histatin-5, the developed material showed “fungal repellent” ability and prevented the formation of biofilm. A similar study was also carried out by Bates et al. who added histatin-5 to denture adhesive material, and observed that the peptide lost its antifungal action, promoting *C. albicans* growth [132]. The authors pointed out that this inactivation may be related to the use of denture adhesive, which may hinder the action of natural peptides present in saliva and responsible for the body′s innate immune response. However, another possibility would be the retention of the peptide inside the matrix of the adhesive, which would block its release and consequently its action. This is possible because, as pointed out by Wen et al., when the histatin-5 layer was formed more inside its material, the antifungal action did not occur. Thus, the best alternative for treatment and prevention of oral candidiasis or denture stomatitis would be the use of systems that allow the release of the peptide or its use as external coating, facilitating its contact with the microorganism and causing its death.

A novel strategy in the development of NPs is their functionalization with a molecule that directs them to the target. This strategy was used by Park et al. who functionalized chitosan NPs with histatin-5 [98]. This peptide is able to penetrate the *C. albicans* cells through an SSa1/2P receptor that directs the NPs to its target. Once in contact with the cell, the NPs release their internal content composed with amphotericin B (AmB), which with histatin-5 causes the death of the yeast. This was the first time that this antifungal peptide was used to functionalize NPs, and the strategy was successful. The peptide was used as a target and also had a synergistic effect with AmB. In addition, AmB showed low cytotoxicity and great efficiency for acting directly on its target. This study shows a new possibility for histatin-5 and its analogues that can be used to functionalize NPs, an area that is still little explored. Some analogues of this peptide have greater antifungal potential such as P113 [133]. This peptide is smaller than histatin-5, and with only 12 amino acids, it is easier to be produced and used to target NPs, which increases its antifungal potential and promotes its action directly on the target cell.

The association of histatins peptides into NPs is still little explored. Most of the new technologies proposed involving the incorporation of this peptide directly into hydrogels and as a coating for dental materials. However, the application of these hydrogels in the oral cavity must be properly studied, as several factors such as salivary flow, swallowing and chewing and the frequent movement of the tongue can quickly remove the product from its place of action, generating a loss of its activity. Another strategy that keeps these peptides in contact with their site of action and protected from enzymatic degradation is the development of liposomes loaded with histatin-5. Liposomes are nanostructured systems formed by the self-organization of phospholipids in bilayers [134]. Histatin-5loaded liposomes were able to preserve the antifungal action of the peptide and control *C. albicans* growth for 72 h. In addition, liposomes were able to release their content for a prolonged time [134]. Although complementary tests are needed, such as system degradation in human saliva and in vivo tests, liposomes have proven to be a suitable system for loading the peptide and to be used in the treatment of oral candidiasis.

As highlighted here, although histatin-1 and histatin-5 have wide potential for oral re-epithelialization, cell migration and adhesion and antifungal activity [127,129,130], there are still problems that must be solved for their appropriate application. Hydrogels produced with chitosan and HPMC are certainly a strategy that solves some problems for the application of these peptides, as well as the coating of dental materials is also a solution to keep the peptide in contact with its place of action. However, there is a lack of studies on the incorporation of these peptides in NPs, which, due to their unique characteristics, are a very attractive tool and helps overcome the barriers for histatin to be used in clinic and therapy.

## 5. Conclusions

As described here, *C. albicans* is a versatile microorganism that can adapt to different environments and colonize the host′s tissue under certain conditions. Some strategies that make this possible are the yeast to hypha transition, the production of proteins that promote its adhesion and invasion to the host cells and the biofilm formation. The Als proteins, specifically the proteins Als5p and Als3p as well as invasin Ssa1p and adhesin Als3p, are important virulence factors that promote cell adhesion and cell invasion, respectively. Besides that, *C. albicans* already shows mechanisms that confer resistance to some classical antifungals like fluconazole. Some of these mechanisms are efflux transporters that do not allow the accumulation of the drug intracellularly. In addition, *Candida* species presents ATP-dependent proteins 1 and 2, and the l proton-motive force-drive protein Mdr1p. Those proteins are related to fluconazole resistance and play an important role in increasing the virulence factors when expressed for the microorganism. Other mutations like increased chitin synthesis and overexpression of the ERG11 are associated with resistance mechanisms to echinocandins and azoles, respectively.

However, the most important and that requires concern are factors that confer resistance to some innate immune response of the human body. The proteolytic cleavage by aspartic proteases and the linkage with the membrane protein Msb2 are an example of resistant mechanisms related to salivary peptide histatin-5. This peptide can be degraded by *C. albicans* extracellular enzymes such as the aspartyl-proteases Sap2p and Sap5p. Beyond that, the polyamine efflux transporters FLU1 can cause the efflux of this peptide and stop its antifungal action. However, there are strategies to avoid these resistance mechanisms and increase the antifungal potential of this peptide and also to promote the escape of these resistance mechanisms. We can highlight here the production of histatin-5 analogs like P113, dh-5, K11R-K17R and others that could be seen in Table 1 in this review. The alteration of their amino acid sequence and their fusion with other peptides are some strategies to avoid this resistance mechanisms shown for *C. albicans*. In fact, there are many works that show the development of new analogs and peptides based on histatin-5, but there are few works that describe their use in clinical treatment or in vivo experiments. The easy degradation by proteolytic enzymes is still a barrier for their applications that must be overcome.

In the light of these observations, we bring here the nanotechnology, specifically the NPs, as a suitable strategy to promote the clinical use for these peptides. Nanoparticles like liposomes, polymeric NPs and chitosan NPs are the most used and have shown great potential for the clinical application of these peptides. Even though more studies are needed, as well as the development of other types of NPs to promote the encapsulation of these peptides, the use of protein delivery systems based in nanotechnology could be a promising way to cope with this limitation and to promote a step forward in the biotechnological field.

## Figures and Tables

**Table 1:** Name and amino acid sequence for the analogues and hybrids peptides based on Histatin-5 primary structure.

Name	Sequence	Reference
dh-5	KRKFHEKHHSHRGY	[111]
dhvar1	KRLFKELKFSLRKY	
dhvar2	KRLFKELLFSLRKY	
dhvar4	KRLFKKLLFSLRKY	[114]
cCF10	LVTLVFV	
cOB1	VAVLVLGA	
cCF10-dhvar4	LVTLVFVKRLFKKLLFSLRKY	
cOB1-dhvar4	VAVLVLGAKRLFKKLLFSLRKY	
P113	AKRHHGYKKFH	[96]
15Hc	ALLHHGLNCAKGVLA	
18Hc	WLNALLHHGLNCAKGVLA	
PH2	AKRHHGLNCAKFH	
HHP1	WLNALLHHGYKRKFH	
WP2	WLNAKRHHGYKCKFH	
Statherin	DSpSpEEKFLRRIGRFGYGYGPYQPVPEQPLYPQPYQPQYQQYTF	[115]
Histatin-3	DSHAKRHHGYKRKFHEKHHSHRGYRSNYLYDN	
DR9-RR14	DSpSpEEKFLRRKFHEKHHSHRGYR	
Hst_4-15_	AKRHHGYKRKFH	[118,120]
Spermidine	GGG-spermidine	
Hst_4-15_-Spd	AKRHHGYKRKFH-GGG-spermidine	
Histatin-5	DSHAKRHHGYKRKFHEKHHSHRGY	[117]
K11R-K17R	DSHAKRHHGYRRKFHERHHSHRGY	
